# Bioresorbable Drug-Eluting Magnesium-Alloy Scaffold for Treatment of Coronary Artery Disease

**DOI:** 10.3390/ijms141224492

**Published:** 2013-12-16

**Authors:** Carlos M. Campos, Takashi Muramatsu, Javaid Iqbal, Ya-Jun Zhang, Yoshinobu Onuma, Hector M. Garcia-Garcia, Michael Haude, Pedro A. Lemos, Boris Warnack, Patrick W. Serruys

**Affiliations:** 1 Department of Interventional Cardiology, Erasmus University Medical Centre, Thoraxcenter, Rotterdam 3015 GD, The Netherlands; E-Mails: carlosacampos1@gmail.com (C.M.C.); takam0401@gmail.com (T.M.); j.iqbal@sheffield.ac.uk (J.I.); 13770668667@139.com (Y.-J.Z.); yoshinobuonuma@gmail.com (Y.O.); HGarcia@cardialysis.nl (H.M.G.-G.); 2 Heart Institute (InCor), University of São Paulo Medical School, Sao Paulo 05403-000, Brazil; E-Mail: pedro.lemos@incor.usp.br; 3 Department of Cardiology, Fujita Health University Hospital, Tokyo 470-1192, Japan; 4 Department of Cardiology, Imperial College London, London SW7 2AZ, UK; 5 Städtische Kliniken Neuss, Lukaskrankenhaus GmbH, Neuss 41464, Germany; E-Mail: michael.haude@uni-due.de; 6 BIOTRONIK AG, Bülach CH-8180, Switzerland; E-Mail: boris.warnack@biotronik.com

**Keywords:** bioresorbable scaffold, drug-eluting stent, bioabsorbable, biodegradable, coronary artery disease, magnesium

## Abstract

The introduction of metallic drug-eluting stents has reduced the risk of restenosis and widened the indications of percutaneous coronary intervention in treatment of coronary artery disease. However, this medical device can induce hypersensitive reaction that interferes with the endothelialization and healing process resulting in late persistent or acquired malapposition of the permanent metallic implant. Delayed endotheliaization and malapposition may lead to late and very late stent thrombosis. Bioresorbable scaffolds (BRS) have been introduced to potentially overcome these limitations, as they provide temporary scaffolding and then disappear, liberating the treated vessel from its cage. Magnesium is an essential mineral needed for a variety of physiological functions in the human body and its bioresorbable alloy has the strength-to-weight ratio comparable with that of strong aluminum alloys and alloy steels. The aim of this review is to present the new developments in Magnesium BRS technology, to describe its clinical application and to discuss the future prospects of this innovative therapy.

## 1. Introduction

The percutaneous treatment of coronary artery disease consists of catheter-based techniques to enlarge lumen of an artery narrowed by an atherosclerotic lesion. The development of this method began in 1977 with balloon angioplasty, which consisted of a mechanical dilatation of the atherosclerotic lesion with the hazard of thrombosis and vascular occlusions due to a combination of elastic recoil and vessel wall dissections (medial and/or intimal) [1]. Furthermore, proliferative neointima and constrictive remodeling could abrogate the transient therapeutic benefit of the dilatation of the stenosis [2]. In 1986, the introduction of metallic stents offered a mechanical solution to the dissection, elastic recoil and constrictive remodeling [3–5]. However, the implantation of these bare metal stents still caused neointimal proliferation leading to in-stent restenosis [6]. A decade later, in 1999, coating and elution of cytostatic and cytotoxic drugs reduced, if not eliminated, the exuberant in-stent neointima in response to the implantation of a foreign body [7–9]. However, this medical device created new enemies: hypersensitive reaction mediated by eosinophils, lack of endothelialization and late persistent or acquired struts malapposition, which are source of late and very late stent thrombosis [10–12].

Considering these historical limitations, the next step in the evolution of percutaneous coronary intervention (PCI) was to create a device capable of dilating the coronary obstruction, providing vascular supports for dissections, preventing elastic recoil and constrictive remodeling, inhibiting neointimal hyperplasia and disappearing “after the job was done”.

Over the last 10 years, considerable efforts have been made to develop fully bioresorbable devices, called bioresorbable scaffolds (BRS). BRS technology has gradually matured, and there are numerous devices available, which are currently undergoing preclinical or clinical testing, and magnesium is an attractive alloy for this concept. The aim of this review is to describe the current concept, the mechanism of absorption and the data available on magnesium-based BRS.

## 2. Potential Benefits of a Transient Scaffold

PCI with BRS has potential advantages over the current generation of metallic bare-metal stent (BMS)/drug-eluting stent (DES) technology. Physiologically, the absence of a rigid metallic cage can facilitate the restoration of the vessel vasomotor tone, adaptive shear stress, late luminal enlargement, and late expansive remodeling. After bioresorption, there would be potentially no triggers for thrombosis, such as uncovered stent struts, durable polymer or remnant drug, with potential reductions in adverse events such as stent/scaffold thrombosis. The absence of foreign material may also reduce the requirement for long-term dual antiplatelet therapy and associated bleeding complications. In the long term, BRS should not hamper future treatment options such as PCI, coronary artery bypass graft, or pharmacological plaque regression [13].

## 3. Magnesium as a Component for BRS

Magnesium is an essential mineral needed for a variety of physiological functions in the human body. The usual daily magnesium intake with a western diet is sufficient to avoid deficiency but seems not to be high enough to establish high normal serum magnesium concentrations that are protective against various diseases. The extracellular magnesium concentration is primarily regulated by the kidney and redundant magnesium cations can be harmlessly and efficiently excreted in the urine [14]. Combining its rapid corrosion with a controlled degradation process through Zinc and Manganese alloying, purification and anodization, magnesium has been developed into a bioresorbable and biocompatible implant material [15].

Although magnesium is available commercially with high degree of purity (exceeding 99.8%), it has low strength and rapid corrosion in unalloyed form. Therefore, it is commonly used in its alloy form, which is possible with a wide variety of elements [15,16].

Although Magnesium is the lightest structural metal, the strength-to-weight ratio of precipitation-hardened magnesium alloys is comparable with that of strong aluminum alloys and alloy steels [16]. Consequently, a magnesium BRS has potential to provide a high radial strength for dilating atherosclerotic narrowing and, hence, higher acute gain of coronary lumen. Another virtue of magnesium as an endoprosthesis is its electrochemical properties. Devices with negatively charged surfaces are less thrombogenic than those with positive surfaces. Magnesium is more electronegative than other metals used for implants and has shown anti-thrombogenic properties *in vivo* [17–21].

## 4. The First Generation Magnesium BRS

**Figure 1.**
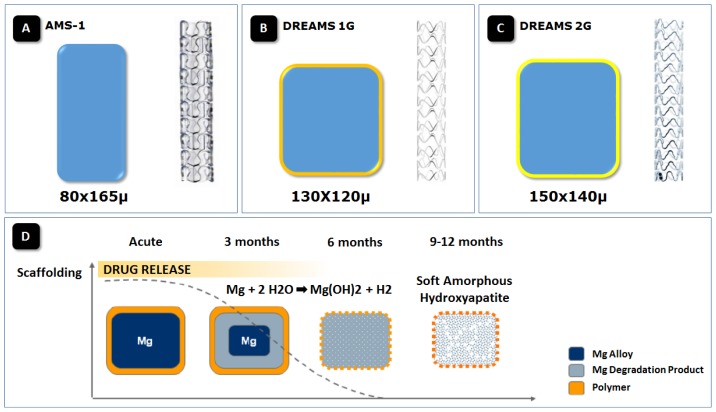
(**A**) Schematic cross-sectional profile of magnesium scaffolds struts of (**A**) uncoated, non-eluting, AMS-1 with 80 × 165 μ; (**B**) DREAMS 1st Generation (DREAMS 1G) with 130 × 120 μ struts and (**C**) DREAMS 2nd generation (2G) with 150 × 140 μ struts. The poly(lactide-co-glycolide)-coating with paclitaxel elution of the DREAMS 1G scaffold is indicated by the thin light orange layer. The PLA-coating with sirolimus elution of the DREAMS 2G scaffold is indicated by the thin dark orange layer; and (**D**) Schematic representation of the resorption process in the drug-eluting absorbable magnesium scaffold. The release of the anti-proliferative drug occurs within the first 3 months after device implantation. Hydrolysis of the scaffold affects the radial strength of the scaffold, resulting in a gradual resorption of the device into a soft amorphous hydroxyapatite at 9 months follow-up. AMS-1, first-generation bare absorbable metal scaffold; DREAMS, Drug-Eluting Absorbable Metal Scaffold.

The first generation of bioabsorbable metal scaffolds (AMS-1; Biotronik AG, Bülach, Switzerland) was made from a WE43 alloy, composed of 93% Mg and 7% rare earth elements (Figure 1). The AMS-1 was a tubular, slotted, balloon-expandable scaffold sculpted by laser from a tube of a bioabsorbable magnesium alloy without drug-elution. The mechanical characteristics of the magnesium scaffolds were similar to stainless steel stents, including low elastic recoil (less than 8%), high collapse pressure (0.8 bar), and minimum amount of shortening after inflation (less than 5%) [22].

In porcine coronary arteries, the histologic evaluation of AMS-1 showed that none of the arteries analyzed had incomplete stent apposition, excess of intimal thickening at the stent edges or intraluminal thrombus. The neointimal tissue proliferation was significantly less in the stented segments of the magnesium alloy scaffold as compared to a control group of stainless steel stents. The reduction of neointima formation was not translated to larger vessel lumen and the overall stented segment was significantly smaller when compared to the stainless steel stent. This can result from underexpansion of the stent at deployment, early or late recoil, or all of the above. Although statistically not significant, the extent of fibrin deposition and inflammation for stented segments of stainless steel stents were slightly higher in the group treated with stainless steel stents than those treated with magnesium BRS. The AMS-1 was largely bioabsorbed into inorganic ions within 60 days of implantation [23].

The AMS-1 was evaluated in a prospective, non-randomized, multicenter, clinical trial (*n* = 63). There were no safety concerns regarding deaths, myocardial infarction, or scaffold thrombosis. However, the long-term patency rates were lower than expected. The in-scaffold late lumen loss (LLL) was 1.08 ± 0.49 mm. The LLL was a combined effect of a decrease in external elastic membrane area (representing 42% of LLL), decrease in scaffold area (18% of LLL) and neointima formation (40% of LLL). Thus, the main mechanism of restenosis (60% of LLL) was a faster than expected scaffold degradation with an early loss of radial force and consequent vessel recoil. The ischemia-driven target lesion revascularization rate was 23.8% after 4 months, and the overall target lesion revascularization rate was 45% at 1 year [22,24–26].

## 5. The Paclitaxel-Eluting Absorbable Metal Scaffold (DREAMS)

To prolong vessel scaffolding AMS-1 was redesigned (Figure 1). The balloon-expandable DREAMS scaffold (Biotronik AG, Bülach, Switzerland) used a refined, slower-resorbable WE43 alloy with 6-crown 3-link design and with a higher collapse pressure than AMS-1 (1.5 *vs.* 0.8 bar). The cross-sectional profile of scaffold struts in DREAMS was redesigned to be square-shaped, as opposed to the rectangular shape in AMS-1. In a porcine coronary model, the scaffold degradation showed a preferential cellularization at the lateral sides of the struts and a square shape slowed the resorption process compared to the first generation magnesium AMS-1 [27]. Thereby, strut thickness was reduced from 165 to 120 μm. To reduce neointimal growth the DREAMS was coated with a 1 μm bioresorbable poly(lactide-*co*-glycolide) polymer matrix (PLGA) containing the antiproliferative drug paclitaxel (0.07 μg/mm^2^) [27].

The first *in vivo* evaluation of DREAMS used a porcine model and evaluated the best lactide to glycolide ratio for the PLGA polymer formulation. This formulation regulates the resorption rate of the drug-carrying PLGA polymer and, therefore, the release of paclitaxel. Seventy-three magnesium scaffolds (ratio of lactide to glycolide of 50/50 in 25 scaffolds, 85/15 with high molecular weight of PLGA in 24, 85/15 with low molecular weight of PLGA in 24) and 36 control stents (18 TAXUS Liberté, 18 eucaTAX) were implanted. The best-performing magnesium scaffold—85/15 with high molecular weight—was equivalent to TAXUS Liberté and superior to eucaTAX regarding late luminal loss, intimal area, fibrin score, and endothelialization. Intimal inflammation score was higher in 85/15H than in the control scaffolds at 28 days, but was similar at 90 and 180 days. Endothelialization was nearly completed within 90 days in all devices [27].

The Figure 1 describes the dynamics and byproducts of magnesium scaffold resorption at 28, 90 and 180 days. Two phases of the resorption process of magnesium alloy were identified. First, a Mg-rich compound containing a large amount of oxygen is formed, possibly representing a mixture of Mg hydroxide and Mg carbonate. Several weeks later, these compounds convert to amorphous calcium phosphate, filling exactly the voids previously occupied by the dissolved scaffold struts. Measured at 28 days, the average *in vivo* degradation rates for the three DREAMS versions ranged from 0.036–0.072 mg/(cm^2^ day) [27].

The first-in-man BIOSOLVE-I trial assessed the safety and performance of this first generation drug-eluting magnesium-based BRS in 46 patients with 47 lesions at five European centers. During the procedure, all devices were successfully delivered. The in-scaffold late lumen loss was reduced at 6 months (0.65 ± 0.5 mm) and at 12 months (0.52 ± 0.39 mm) compared to 1.08 ± 0.49 mm of the prior generation bare AMS-1 magnesium scaffold. However, the late lumen loss with DREAMS still did not match the excellent results of currently available drug-eluting stents.

**Figure 2.**
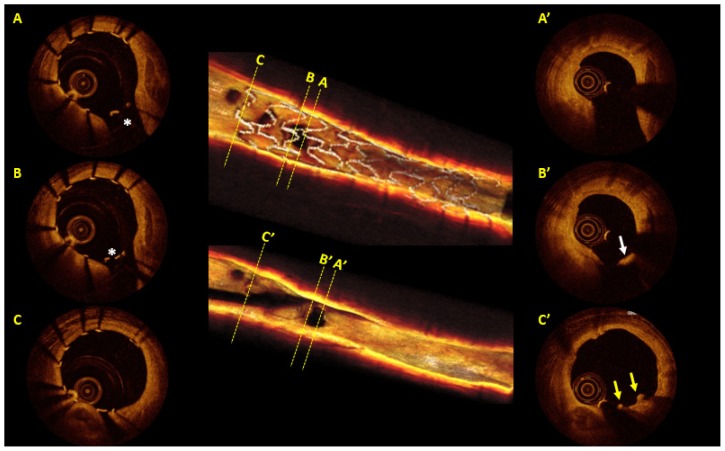
Post-implantation and 12-month follow-up optical coherence tomography (OCT; LightLab Imaging, Westford, MA, USA) of a percutaneous coronary intervention of the left anterior descending coronary artery, whereby a 3.25 × 16 mm paclitaxel-eluting absorbable metal scaffold (DREAMS 1G; Biotronik, Bülach, Switzerland) was implanted. Post-procedurally a side branch was jailed by the struts of DREAMS 1G (Panels **A** and **B**). At 12 months follow-up, OCT showed a smooth luminal surface with moderate neointimal hyperplasia in the scaffolded segment. Just few remnants of struts were still visible with shadows (panel **C’**, yellow arrows). The struts overhanging a side branch ostium were partially replaced by a neointimal membranous bridge (panel **B’**, white arrow), while three-dimensional OCT revealed unobstructed and widely opened ostium of side branch.

In the BIOSOLVE-I trial, data for serial OCT were available for only seven patients with 5791 assessable struts. At 6 month follow-up, 97.2% (95% CI_96.7–97.6_) of the struts were apposed and at 12 months 99.8% (95% CI_99.6–99.9_) of the struts were apposed with only 0.1% (0.03–0.3) persistent incomplete strut apposition and 0.1% (0.03–0.3) late acquired incomplete strut apposition. An illustrative case of DREAMS BRS with OCT images is shown in Figure 2. At 12 months, three of 43 (7%, 95% CI_1.7–19.3_) patients had target lesion failure with no cardiac death or scaffold thrombosis [28].

## 6. The Sirolimus-Eluting Absorbable Metal Scaffold (DREAMS 2nd Generation)

**Figure 3.**
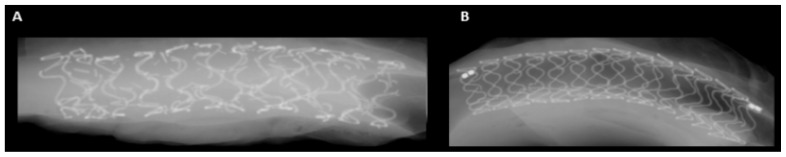
High-resolution faxitron evaluation from a porcine coronary model after 90 days of implantation. At this time point, faster dismantling rate and resorption of the scaffold DREAMS 1G (**A**) than its latest development, the DREAMS 2G (**B**) could be detected.

DREAMS was further modified to create the next generation: the DREAMS 2nd generation (DREAMS 2G, Figure 1) which is made of a WE43 alloy with 6-crown 2-link design and a strut thickness of 150 μm with radiopaque markers at both ends (made from tantalum) resulting in slower dismantling and resorption rate (Figure 3). The distal markers were added to make scaffold implantation and possible post-dilation more precise. To further reduce the neointima formation, the DREAMS 2G was coated with a bioresorbable polylactic acid polymer (7 μm) featuring sirolimus at a dose of 1.4 μg/mm^2^—known to have more potent antiproliferative effect than paclitaxel [29–31]. DREAMS 2G has completed preclinical assessment and is currently being evaluated in BIOSOLVE-II trial.

## 7. Conclusions

BRS is a relatively new technology introduced to address the limitations of the traditional metallic stents. BRS will usher the practitioner in a new era of treatment of coronary lesions, as they provide temporary vessel scaffolding and then disappear, thereby allowing for the restoration of the vessel wall physiology and vasomotion. Magnesium alloy has a great potential since it is a metal with high radial strength and less thrombogenic electrochemical properties than most metals used for implants. Evidence from studies of the DREAMS 1st generation indicates that it has improved the drawbacks of the first generation bare AMS-1 (e.g., rapid bioresorption and device shrinkage). The DREAMS 2nd generation has completed its preclinical assessment and the experimental data suggest that it may be able to compete with the drug-eluting metallic stents in terms of safety and efficacy. BIOSOLVE-II trial will test the real potential of this technology.
